# Big Data Workflows: Locality-Aware Orchestration Using Software Containers

**DOI:** 10.3390/s21248212

**Published:** 2021-12-08

**Authors:** Andrei-Alin Corodescu, Nikolay Nikolov, Akif Quddus Khan, Ahmet Soylu, Mihhail Matskin, Amir H. Payberah, Dumitru Roman

**Affiliations:** 1 Department of Informatics, University of Oslo, 0373 Oslo, Norway; alin.corodescu@gmail.com; 2 SINTEF AS, Software and Service Innovation, 0373 Oslo, Norway; nikolay.nikolov@sintef.no; 3 Department of Computer Science, Norwegian University of Science and Technology, 2815 Gjøvik, Norway; akif.q.khan@ntnu.no; 4 Department of Computer Science, OsloMet—Oslo Metropolitan University, 0166 Oslo, Norway; 5 Department of Computer Science, KTH Royal Institute of Technology, 114 28 Stockholm, Sweden; misha@kth.se (M.M.); payberah@kth.se (A.H.P.)

**Keywords:** big data workflows, orchestration, data locality, software containers

## Abstract

The emergence of the edge computing paradigm has shifted data processing from centralised infrastructures to heterogeneous and geographically distributed infrastructures. Therefore, data processing solutions must consider data locality to reduce the performance penalties from data transfers among remote data centres. Existing big data processing solutions provide limited support for handling data locality and are inefficient in processing small and frequent events specific to the edge environments. This article proposes a novel architecture and a proof-of-concept implementation for software container-centric big data workflow orchestration that puts data locality at the forefront. The proposed solution considers the available data locality information, leverages long-lived containers to execute workflow steps, and handles the interaction with different data sources through containers. We compare the proposed solution with Argo workflows and demonstrate a significant performance improvement in the execution speed for processing the same data units. Finally, we carry out experiments with the proposed solution under different configurations and analyze individual aspects affecting the performance of the overall solution.

## 1. Introduction

In recent years, harnessing large data sets from various sources has become a pillar of rapid innovation for many domains such as marketing, finance, agriculture, and healthcare [1]. The big data domain has evolved rapidly, and new challenges have arisen at different levels of the technological stack, from the complex business logic to the infrastructure required to process the ever-increasing volume, velocity, and variety of data. Working with big data is a complex process involving collaboration among a wide range of specialisations (such as distributed systems, data science, and business domain expertise) [2,3,4]. Handling such complexity naturally comes with an increased cost, and the value extracted from the data must, thereby, offset this cost.

Big data workflows formalise and automate the processes that data go through to produce value by providing necessary abstractions for defining workflows and efficiently leveraging underlying hardware resources. In the context of big data workflows, we consider computing resources, such as processors (CPUs), memory, and storage, as relevant hardware resources (from now on referred to as just resources) among others, e.g., [5,6]. Big data workflows usually integrate various data sets and leverage different programming languages or technologies to process data. Therefore, a desirable feature of a big data workflow system is to orchestrate workflows in a technology-agnostic manner, both in terms of data integration and processing logic. Accordingly, approaches based on software containers have emerged to create and execute workflows using processing steps in line with these considerations. While it is beneficial to leverage software containers, packaging and isolating applications for deployment, to better separate concerns in a big data workflow system, higher-level abstractions come with a performance penalty; thus, it becomes more relevant to ensure the system performs as efficiently as possible.

Traditionally, cloud service providers have been the standard solution for working with big data. However, cloud services are inherently centralised in a small number of locations (i.e., data centres) worldwide. Moreover, with the advent of the Internet of Things (IoT), significant amounts of data are generated at edge networks [7]. With the data processing happening on geographically distributed systems across edge and cloud resources, reducing the delay and cost of transferring data over the network becomes crucial. Transferring massive amounts of data to the cloud is expensive and may incur latency, making low-latency scenarios unfeasible. To address these issues, the edge computing paradigm [8] aims to complement cloud computing by leveraging hardware resources situated closer to the edge of the network to offload processing, reduce transfer cost, and satisfy the latency requirements. However, existing solutions are mainly designed for cloud-only workloads, making them unsuitable or inefficient for workloads spanning both the cloud and edge. In this context, in order to alleviate the aforementioned problems, this article addresses the following research questions:

- How can data locality be implicitly integrated with container-centric big data workflow orchestration?
- How can software containers be used to facilitate the interaction with different data management systems as part of big data workflows?
- How can containers encapsulating processing logic be used to improve the performance of big data workflows?

To this end, we propose a novel architecture for container-centric big data workflow orchestration systems that makes containerised big data workflow systems more efficient on cloud and edge resources [9]. Our proposed approach considers (i) data locality, (ii) leverages long-lived containers (i.e., containers whose life-cycle is not tied to a particular data unit) to execute workflow steps, and (iii) handles the interaction with different data sources through containers. We compare our proposed approach with a similar existing solution, Argo workflows. The comparison shows that by considering data locality, our proposed approach significantly improves the performance in terms of data processing speed (up to five times better). We also present a set of experiments with different configurations analysing individual aspects affecting the performance of the overall solution.

The rest of the article is structured as follows. Section 2 gives the relevant context. Section 3 provides an analysis of the problem. Section 4 discusses related work. Section 5 presents the proposed solution and Section 6 describes its implementation. Finally, Section 7 provides experimental evaluation of the proposed solution, and Section 8 concludes the article and outlines the future work.

## 2. Background

Big data has quickly gained momentum as it allows the exploitation of data to uncover hidden trends that give valuable insights that drive business decisions and support research (e.g., [10,11]). Big data solutions have been successfully leveraged in a large number of industries. The general applicability of big data patterns stems from the fact that many real-world phenomena can be better understood and predicted given sufficient data. The characteristics, e.g., volume, velocity, and variety of big data, translate into challenges at all levels of the technology stack for big data processing:

1. At the infrastructure level, significant raw network, storage, memory, and processing resources are required to handle big data. Often these resources are provided by multiple machines, organised in a distributed system.
2. At the platform level, software frameworks that can effectively leverage the available resources and handle the ever-changing needs of big data operations need to be continuously developed.
3. At the application level, algorithms running on the previously mentioned platforms need to be devised and combined to extract value from the data. Applications can also facilitate the interaction with a big data solution (e.g., visualisation tools used by business executives to analyse the results produced by the solution).

Devising algorithms and tools that help process and extract value from the data further amplify the complexity and cost of building comprehensive big data solutions. Consequently, another V, the value the solution generates, which needs to offset the high cost, is often included as a central characteristic of big data.

### 2.1. Big Data Workflows

**Figure 1:**
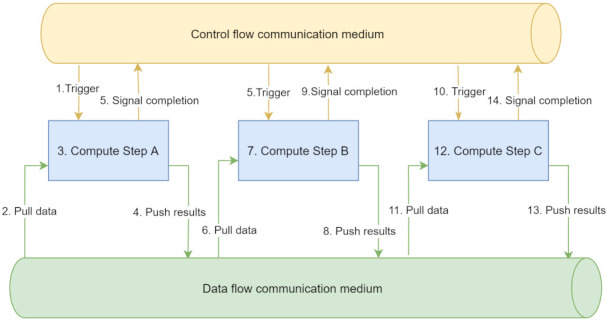
Workflow as sequence of steps with communication mediums.

Raw data must be taken through a series of operations (e.g., cleaning, aggregation, and transformation) before producing valuable insights. While it is possible for each of these steps to be manually triggered and independently controlled in an ad-hoc manner, workflow orchestration tools facilitate the automation of the execution and sequencing of these operations, allowing reliable and reproducible execution of complex processes. Key concepts used in workflow orchestration [12] (see Figure 1) include:

- **Step:** Steps are the atomic units upon which workflows are defined. A step encapsulates business logic units that receive data as input from a data source, processes them, and then pushes the outputs to a data sink.
- **Workflow:** Individual steps can be linked together to form a workflow. A workflow is a linear sequence of steps with the semantics of executing the steps. Big data workflows are specified in more complex configurations. A widely used model stems from graph theory, which describes a workflow as a directed acyclic graph (DAG).
- **Data communication medium:** The distributed nature of big data processing warrants the existence of a data communication medium through which information can be exchanged between the components of the system. Thereby, both the inputs and the outputs of a step connect to such channels.
- **Control flow communication medium:** To be able to execute the workflows, control messages (e.g., triggering a step, notifying when a step has finished) have to be exchanged within the system through a control flow communication medium. Examples of such communication mediums include point-to-point network communication between components and message queues.

Creating big data workflows is a complex process involving knowledge from multiple domains (hardware provisioning, cluster management, data handling, different processing steps, definition of workflows according to business needs, and orchestration). Delegating the different responsibilities to independent components allows easier development of both the orchestration frameworks and the workflows running on top of them, reducing the costs and making big data workflows more accessible.

### 2.2. Cloud and Edge Computing

Cloud computing paves the way for accessible, affordable, and efficient big data processing through scalable, elastic, and “pay-per-use” models. Cloud deployments are best suited for cases where the producer of data is mainly centralised. However, with the advancement of ubiquitous computing, tremendous amounts of data are produced by devices (e.g., sensors, personal devices, and vehicles) at the edge of the network. With the number of such devices increasing rapidly, the traditional model for using centralised cloud resources becomes infeasible. Edge computing [8] complements cloud computing by performing computations on resources physically located closer to the edge of the networks. In this respect, Computing Continuum [13] refers to all available resources for a system, from the edge of the network to the cloud.

Although the edge computing paradigm addresses some fundamental limitations of cloud computing, it also faces a different set of challenges [14]. Among others, these notably include:

- Hardware resources on edge devices exhibit different characteristics and capabilities compared to cloud resources (e.g., processor architectures, processor clock speeds, and amounts of memory). Therefore, software running on such devices has to be designed to consider these resource constraints. At the same time, edge deployments offer limited or no elasticity.
- Edge resources are geographically distributed, and the latencies can differ significantly depending on the distance between the communicating parties.
- Geographical distribution also raises logistical challenges, as these devices can be spread over wide areas and sometimes even in hard-to-reach locations, making provisioning and maintenance a significant challenge.
- The nature of edge resources also makes them prone to failures at the device level (hardware failures) or supporting infrastructure (network failures). Software solutions targeting edge deployments need to tolerate failures gracefully and, if possible, operate offline for extended periods.
- Security and privacy are two complex domains where edge computing plays a significant role. On the one hand, processing data closer to the source can make it easier to adhere to a certain jurisdiction and ensure better security and privacy. On the other hand, large-scale edge deployments are inherently more complicated to secure, mainly due to their geographical spread, and the risk of having devices compromised through physical interference is much higher than in a cloud-only setup.

### 2.3. Software Containers and Big Data

Software containers are standardised, isolated units of software that package everything required to run an application (https://www.docker.com/resources/what-container, accessed on 8 November 2021). They provide a lightweight and faster virtualisation alternative to hypervisor virtualisation [15,16]. Software containers exhibit a series of characteristics that make them applicable to a wide range of domains:

- Containers ensure the packaged software runs in complete isolation from other applications on the same operating system. Packaging all dependencies in a container can avoid challenging issues such as conflicting dependencies and complex prerequisite configurations.
- The low overhead introduced by containers allows many containers to be run efficiently on a single node, making them a good fit for resource-constrained environments.
- Software that can run in a container is not limited to a particular technology or programming language, allowing solutions leveraging software containers to orchestrate cooperation of components developed using different technologies easily.
- Containers are a widely adopted standard for software packaging, which translates into two major benefits. First, containerised software is easier to share and reuse across different environments. Second, containers can be used to move the execution of logic onto a distributed system’s nodes (“function shipping”).

Container orchestration solutions, such as Kubernetes (https://kubernetes.io/, accessed on 8 November 2021), simplify the deployment and management of highly distributed systems by creating abstractions for the underlying infrastructure and facilitating the interaction between the components of an application. Software containers are extensively leveraged in cloud environments [17] and, in some cases, containerised applications are referred to as cloud-native applications [18]. Several works also identify software containers as a feasible technology for resource-constrained edge environments [19,20,21]. In the context of big data, containers have been used to simplify the deployment and management of entire big data solutions or individual components. Leveraging containers in big data solutions can also lead to performance improvements when compared to the hypervisor-based virtualisation alternatives [22].

There is a distinction between the two strategies of using containers in big data solutions at a high level. First, software containers are leveraged to deploy and manage the components of a big data processing platform. Although this approach simplifies the deployment process, it does not influence the run-time aspects of the platform. Second, software containers are used as an integral part of the architecture and as a mechanism through which custom behaviour can be injected into the platform (e.g., data processing logic). Software containers have gained much traction in the field of microservices as they greatly simplify the management of highly distributed systems [23,24]. Such an architecture can provide some benefits, including modularity, loose coupling, and technology independence, which align with the needs of big data workflow systems.

## 3. Problem Analysis

The high velocity of the data, combined with the large volume, mandates the processing to happen efficiently and cost-effectively to produce value that outweighs the costs. Therefore, execution time and bandwidth usage are two indicators that are often measured in big data systems and determine the feasibility of a given system. In the following, we introduce the essential concepts and techniques to reduce the execution time and bandwidth usage in big data workflows.

### 3.1. Data Locality

Data locality [25] refers to moving computation closer to the data, which is typically cheaper and faster than moving data closer to the computation. The nature of working with big data mandates the resources (e.g., network, memory, CPU, disk) of multiple machines to be pooled together in a distributed system. A desirable characteristic of distributed systems is to hide the complexities of the distributed resources behind a single interface, such that the entire system appears as a single entity (e.g., cloud storage systems such as Amazon S3). However, this makes it more challenging to leverage individual hosts comprising the distributed systems. For example, a fundamental invariant of current computer architectures is that a CPU can only work with data present in the memory of the same machine.

Consequently, the movement of data across machines becomes an integral part of any big data system. With traditional communication protocols that rely on the operating system network stack (e.g., TCP/IP-based protocols), latency becomes critical for many use cases. To this end, more efficient protocols have emerged. For example, RDMA (Remote Direct Memory Access) [26] is a protocol that allows the transfer of data stored in the memory of one machine to another without involving the CPU or the operating system kernel through specialised network cards. As the volume of data is significant in the context of big data, the network traffic and the associated latency of transferring data between machines can influence the overall cost and performance. Even for solutions targeted at centralised deployment (such as cloud deployments), data locality has proven to be effective in reducing the cost and execution times [27,28]. For example, Apache Spark [29] leverages the information provided by the Hadoop File System (HDFS) and knowledge about outputs of previous executions of jobs to minimise the data transfer.

One of the core motivations of the edge computing paradigm is reducing the amount of data transferred from the edge of the network to the cloud and supporting lower latency scenarios, making data locality a primary concern for any edge computing solution. However, data locality is only one aspect that can be considered when scheduling data processing tasks. Other aspects such as load distribution and heterogeneity of the available resources on different nodes need to be balanced together with data locality to perform the tasks effectively [30]. Studies exist that propose advanced scheduling strategies to balance the reduction in data transfer with load distribution (e.g., [31,32]).

### 3.2. Inter-Component Communication Optimisation

Separation of concerns and delegating responsibilities to different components have numerous benefits; however, the communication between components may introduce other performance and efficiency overhead due to message serialisation and transfer through potentially slow mediums. For example, a simple method invocation in a monolithic solution can be turned into a REST API call for a solution where components are separated. The choice of communication protocols directly impacts the performance and bandwidth utilisation, as different protocols provide different guarantees related to data transmission (e.g., TCP ensures an ordered and lossless transmission but requires multiple round trips to establish connections and exchange data, while UDP is faster but less reliable).

Different protocols introduce additional overhead by injecting more data in transmission packets (e.g., HTTP headers). Techniques, such as compression and binary serialisation, help reduce the size of the payload. Furthermore, there exist studies exploring the use of RDMA-backed memory channels to support fast and efficient inter-container communication (e.g., [33]). Apart from the bandwidth utilisation and speed of a particular protocol, the contract defining the communication between two entities (message format, content, and semantics) plays a significant role in facilitating the integration between components. Defining and enforcing a communication contract between components allows decoupling them from one another.

### 3.3. Lifecycle Management of Containers

Software containers are a lightweight virtualisation alternative to traditional hypervisor-based virtualisation; however, there is still a cost associated with starting up and shutting down containers on demand. Life-cycle management has a high impact on workflow execution time, and reusing containers to process multiple units of data (i.e., long-lived containers) is a way to improve it [34]. For many use cases, the execution time of the work delegated to a particular container is high, thus making the overhead of instantiating containers negligible.

In edge computing environments, the available resources are limited, and less data can be processed on a single host at a given point in time. Furthermore, with data being constantly processed in small batches (or even streamed), there is a need for this processing to happen as quickly as possible to achieve the desired throughput. In such cases, the overhead of setting up and tearing down containers can quickly add up and become a significant bottleneck for the solution’s performance.

### 3.4. Integration with Data Management Solutions

One of the pillars of big data processing is to reason over and process heterogeneous data sets together in a unified manner. These data sets can be stored using different technologies, and the interaction with these technology requires complex logic in itself. Thus, big data workflow systems should facilitate easy integration with different data management solutions, such as databases, file systems, cloud storage, and Web services.

## 4. Related Work

In this section, we first present and discuss the related work in terms of existing published literature and then in terms of existing tools.

Regarding the published literature, Valerie et al. [35] discuss the effect of in-memory processing and data locality on neuroimaging data sets and show the importance and benefits of data locality. However, they do not propose any new system or orchestrator tool and only evaluate the performance of existing systems. Ching et al. [36] propose new techniques for implementing locality-aware virtual machines. They mainly focus on improving the performance of MapReduce programs in heterogeneous and hybrid cloud environments and propose a technique to enhance data locality using a virtual machine mapping technique. Thereby, the locality-aware technique balances workloads and reduces communication overheads at run-time, but it is only restricted to MapReduce applications. Therefore, it cannot be employed with the more general-purpose software containers. August and Christoph [37] present a method of extending smart containers for data locality-aware skeleton programming. They extend the existing SkePU skeleton programming framework to enhance the performance of sequences of transformations on smart containers. However, the framework does not provide support for orchestrating workflows or container lifecycle management.

Bu et al. [38] describe a task scheduling technique for MapReduce applications that minimizes interference while keeping task-data localization. The study, however, disregards network effects, assuming that data flow between co-hosted virtual machines is equally efficient as local data access. Choi et al. [39] present a mechanism for locality-aware resource management in High-Performance Computing (HPC) cloud environments, called Data-Locality Aware Workflow Scheduling (D-LAWS). Their solution condenses virtual machines and includes task parallelism through data flow into the task execution planning of a data-intensive scientific process. However, they do not take into account the role of software containers in scientific workflows.

Ahlehagh et al. [40] present a video-aware scheduling strategy that includes storing video data in a macro-base station to boost video throughput and lessens the likelihood of movies freezing. A heuristic approach to the storage allotment issue in macro-base stations is presented by Gu et al. [41]. Small base stations may deliver better data rates than macro-base stations since they are located closer to users. Finally, Vengadeswaran and Balasundaram [42] propose an approach that also considers the data locality factor, but it is limited to Hadoop. The default data placement strategy of Hadoop creates and allocates blocks randomly across the cluster. To overcome this issue, they propose an optimal data placement strategy to improve the performance of big data applications. These methods focus on optimising data placement strategies rather than the real-time migration of computing steps closer to the data.

In the following, we review existing orchestration tools selected according to the following criteria: (i) ability to incorporate data locality in the orchestration process, (ii) support for container lifecycle management, and (iii) the ease of integration with data management solutions.

- Snakemake [43] is a workflow orchestration tool that supports wrapping individual steps in containers, and different data solutions can be integrated into workflows by extending the Snakemake codebase. However, there is no support for controlling where the computation happens (data locality).
- Kubeflow (https://www.kubeflow.org, accessed on 8 November 2021) is a workflow orchestration tool for machine learning-related workflows. The only storage supported is Minio (a cloud-native, open-source storage solution implementing Amazon S3 cloud storage protocol). It offers no support for data locality.
- Makeflow [44] is a workflow orchestration tool that can orchestrate workflows on a wide variety of infrastructures. However, it does not have any built-in support for different data management systems or data locality features.
- Pachyderm (https://github.com/pachyderm/pachyderm, accessed on 8 November 2021) is another machine learning workflow orchestration solution, but the only storage system it supports is the Pachyderm file system, a distributed file system built to provide supporting features to Pachyderm.
- Pegasus (https://pegasus.isi.edu, accessed on 8 November 2021) is a workflow orchestration solution that supports containerised steps and leverages the location of the processed files to schedule the steps on the same host. However, its data management is limited to using only file systems.
- Airflow (https://airflow.apache.org, accessed on 8 November 2021) is one of the most popular data workflow orchestrators that supports the execution of the workflows on a Kubernetes cluster. It also controls where instances of steps are created, but it should be set manually when the workflow is defined, making it inefficient in dynamically-changing environments. It is possible to integrate different data management solutions by extending the Airflow code with providers, limiting it to Python implementations only.
- Argo Workflows (https://argoproj.github.io/projects/argo, accessed on 8 November 2021) is a workflow orchestration solution natively built on Kubernetes and supports data locality through a set of mechanisms. Similar to the Airflow solution, different data management solutions can be integrated but require ad-hoc changes and integration with the Argo code libraries.

All of the considered solutions leverage short-lived containers as part of the orchestration—a container is created to execute work and is destroyed as soon as the processing completes because these solutions target primarily batch processing scenarios. In terms of data locality specification, Argo offers the most expressive features as it leverages the complete functional offering of Kubernetes. However, by default, the limitation of having to specify data locality at workflow definition time (introduced with Airflow analysis) applies to Argo. Argo offers a mechanism through which respective outputs of processing steps can be used to modify the parameters (for data locality in this case) of subsequent steps in the workflow, allowing for dynamic data locality configurations at run-time. However, such an approach would require additional logic to be injected into the processing step. Although limited in terms of data locality features, Pegasus does handle data locality implicitly, without modifying the workflow definition. In contrast, for both Argo and Airflow, while offering more expressive data locality features, the workflow definition has to capture these details, thus breaking the separation of concerns principle.

## 5. Proposed Solution

We propose an approach based on a workflow system architecture covering the run-time considerations of big data workflows that take into account the separation of concerns principle. The proposed architecture has three main layers:

1. **Control layer:** It is responsible for the execution of workflows concerning their definitions (e.g., correct step sequencing and data being processed). The main component of the control layer is the **orchestrator**.
2. **Data layer:** It collectively refers to all the components involved in data handling (i.e., storage and retrieval of data, and moving data between hosts to make it available to compute steps that require it). The layer includes the **data store** component, referring to the technology used to store data (e.g., distributed file system and cloud storage) and the **data adapter** component, serving as an interface between the data store and the other components in the workflow.
3. **Compute layer:** It refers to the processing logic contained in the steps used in the workflow. The compute layer is composed of multiple **compute steps**, and, in a sequence, they form a workflow. Additionally, the approach allows that multiple instances of the same compute step type run in parallel.

The components of the different layers can be spread across multiple hosts, and the orchestrator serves as the coordinator of the centralised architecture. Using a centralised architecture is motivated by leveraging data locality when the execution of big data workflows requires knowledge about the entire system (e.g., component physical placement) and the data that flows through it (the physical location where data are stored). The centralised architecture greatly simplifies the acquisition, management, and usage of this information. Data are organised into discrete, indivisible, and independent units when passing through the system. These represent the units of work at both the orchestration level and individual step level. Each unit is processed independently, and multiple units can be processed in parallel across different steps. Handling the execution of the workflow at the data unit level can improve the performance significantly compared to the models that execute steps synchronously (all outputs of the previous step have to be available to start the next one) [12,45].

**Figure 2:**
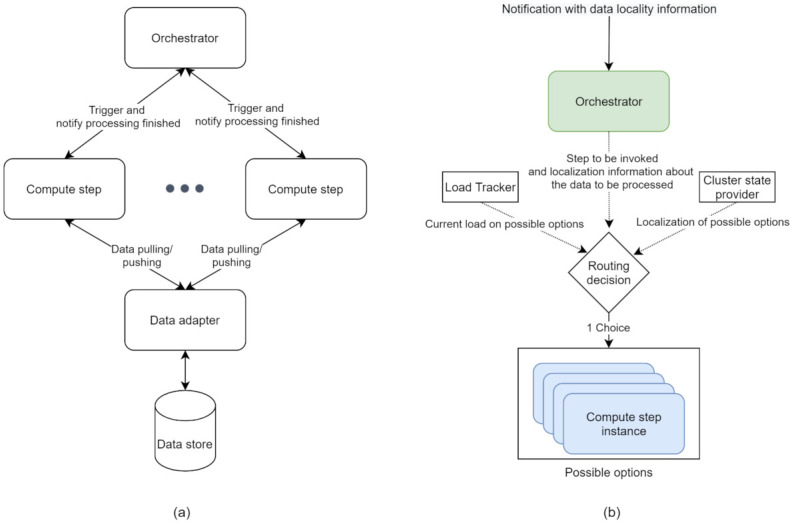
(**a**) Detailed view of a compute step, and (**b**) data locality as a multi-objective optimisation problem.

Whenever a new unit of data is available in the system, the orchestrator is notified, and it passes on the notification to a “compute step” for processing. The compute step may produce one or more outputs from the input, each being a new data unit that continues to flow through the system independently of the others. Organising the work in independent units enables tasks to be distributed across all the available resources, allowing the proposed solution to scale horizontally (increase the processing power by adding more hosts in the distributed system). Figure 2a depicts a high-level overview of the three layers and their interactions. The following sections present each layer in detail.

### 5.1. Control Layer

Upon receiving a notification indicating that new data are available to be processed, the orchestrator needs to determine what type of compute step needs to invoke for the current data unit, according to the workflow specification. For example, in a workflow consisting of three sequential steps, the data units outputted by the second step need to be passed to an instance of the third step. Throughout the system, there can be multiple instances of the same compute step type. The orchestrator is responsible for choosing one of the instances to process the data.

Instead of relying on traditional load balancing algorithms (such as round-robin, random, and least connections), the orchestrator needs to employ a custom routing decision algorithm that takes the number of variables as input. The orchestrator decides about the routing based on available information, such as data locality, current load, varying resource availability, cost, and existing policies (see Figure 2b). This list is not exhaustive and presents only a subset of potential aspects considered when making a routing decision. Optimising cost and performance while ensuring all the explicit requirements of the workflow are met makes the routing a complex, multivariate optimisation problem, with no clear best decision where trade-off in one or more areas is necessary.

To provide data locality, the orchestrator needs to know the physical location of both the data to be processed and the possible target step instances and also a model to calculate the distance between two locations. Moreover, the orchestrator needs to continue operating when presented with unexpected, partial, or missing locality data, as some data adapters may not provide granular data locality information. The inputs to the routing decision need to be acquired and made available by the module responsible for the decision. Depending on the volume and velocity of the units of data flowing through the orchestrator, the calculation of the routing decision needs to be efficient to avoid spending a significant amount of time routing each unit. This means that some of the inputs may have to be pre-calculated or estimated asynchronously, as acquiring information about all the possible targets synchronously may incur a significant performance cost.

### 5.2. Data Layer

The ability to share data between the nodes hosting the compute step instances is a fundamental requirement of the proposed solution. This allows the steps to pass data from one another as part of the workflow execution. All the interactions with the data storage happen through the data adapter in the proposed architecture, serving as an intermediary between the data storage technology and the orchestration components. The data adapter model hides the complexities and particularities of interacting with the data storage by separating the logic into a dedicated component that exposes a simplified interface for external communications. Separating data handling concerns from the compute step creates a modular architecture that facilitates and encourages the reusability of both components in multiple workflows. For example, a compute step processing images can be used to process images from multiple data sources, and the same data solution can be reused with different compute steps.

In addition to reusability, separating the compute steps from the data adapter allows for more flexibility in terms of technologies chosen to implement either of them. For example, a compute step can be implemented in Python while the data adapter in Java and the two components can still communicate. Apart from interacting with the underlying storage, the data adapter is also responsible for providing the other components in the system with information about the physical localisation of the data to support locality-aware work scheduling. The data locality model employed to communicate the localisation information needs to have the following characteristics:

1. It needs to apply to both the data units flowing through the system and the compute step instances, as the information it captures is used to route data units to be processed on compute step instances in proximity.
2. The localisation information needs to apply to resources throughout the Computing Continuum, and a distance measure needs to be determinable for any two localisations.
3. The data locality model needs to be granular enough to capture host-level information.
4. Different data storage solutions have different capabilities of exposing information about where data are stored; thus, the data locality model should support reserved values to indicate that parts of the information are missing.

### 5.3. Compute Layer

The compute steps follow a simple execution model since both the orchestration and data handling logic are handled by external components:

1. A compute step is provided with a unit of data as input (provided by the data adapter).
2. The processing logic is applied in the compute step.
3. The processing logic can produce one or more outputs, picked up by a data adapter, resulting in notifications for the orchestrator.

This architecture gives complete freedom to execute any logic that can be run in containers as the implementation of the compute step has no restrictions over what the processing logic can do with the input data. Neither the input nor the output data are typed.

### 5.4. Extension Model

A widely used model for building upon and extending a framework is integrating a software development kit (a set of language-specific libraries) that handles the interaction between the features provided by the framework and the code extending or building upon it (user logic). The communication between the two components then happens via language-specific constructs, such as method invocations, and is backed by the memory of the hosting process. With container technologies easing the management and deployment of microservices, the framework functionality can be completely separated from the user logic by being placed in a dedicated container. This container can communicate with the user logic (presumably in another container) through an external communication medium.

**Figure 3:**
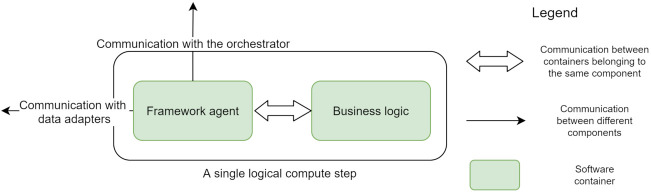
A detailed view of a compute step.

This approach provides better isolation of the two components and allows for integration between different programming languages or frameworks, as the communication is agnostic to the used programming language. Even though the communication medium (e.g., network calls, files, sockets) can be accessed by both components, a crucial aspect is to ensure the communication protocol (e.g., HTTP, gRPC) is supported by both ends. The current architecture opts for the container-based extension model as it aligns better with the architecture requirements. The container-based extension model becomes apparent when diving deeper into the architecture of a compute step (Figure 3). A compute step is logically composed of a **framework agent** and processing logic, running in a separate container. The framework agent container is responsible for coordinating the execution in the context of a single step (retrieving the input data, triggering the processing logic, handling the output data). It effectively hides the complexities related to the orchestration and acts as the intermediary between the data and compute components. Thus it allows them to have simplified interfaces they need to adhere to dedicated only to their function (handling data or processing it), as follows:

1. The framework agent needs to accept requests from the orchestrator to process a unit of data.
2. Based on the instructions received from the orchestrator, it reaches out to the data adapter to retrieve the input data.
3. Once the input data is accessible to the container hosting the processing logic, the agent must send a request to trigger the computation.
4. The output data are passed to the data adapter and the orchestrator is notified that new data have become available.

By leveraging the container-based extension model, the microservice-inspired architecture combines the code contributed by the user (i.e., data adapter, business logic, and data store) and the framework provided components (i.e., orchestrator and framework agent) to define and execute workflows. By injecting two components implementing simple interfaces (one for data handling and one for processing logic), the framework can orchestrate the execution workflows composed of steps implemented in different programming languages or technologies and leverage different data storage solutions. The data handling and processing logic are completely separated, allowing them to be created and to evolve independently, thus helping with the separation of concerns between the stakeholders involved in creating workflows. The proposed architecture allows the framework users to inject data and processing logic into a workflow definition, combining the two elements.

## 6. Implementation

We implemented our proposed solution as a proof of concept using Docker and Kubernetes [15]. The code of our proposed solution, along with the associated Dockerfiles, and Kubernetes YAML files to deploy the model to Kubernetes clusters, are publicly available under the MIT license (https://github.com/alin-corodescu/MSc-workflows, accessed on 8 November 2021).

Docker is used for building the container images for both the framework components (orchestrator and framework agent) and the examples of pluggable components (compute steps and data storage). The container images contain all the information needed by a container run-time to run the components. At run-time, the container orchestration solution uses the images to instantiate the different components as needed. Kubernetes was used to manage and orchestrate the deployment and communication between the components. Kubernetes is the industry standard for container orchestration and exposes abstractions over the hardware resources it manages. Additionally, it is tailored to work well in resource-constrained environments, such as edge environments.

The following is a brief introduction to the main Kubernetes abstractions and features referenced in the remainder article:

1. **Nodes:** These are the abstraction Kubernetes uses for the hosts making up a cluster. These can either be physical or virtual machines.
2. **Pods:** These are the smallest units that Kubernetes manages and deploys. A pod is a group of one or more containers, logically belonging together to perform a particular task.
3. **Services:** These are a type of Kubernetes resource that helps connecting pods or expose functionalities outside the cluster. A selection criterion is used to identify the pods hosting the application the service exposes. The communication with the service is done through a designated IP address and port, with the request routing and load balancing between the pods being handled by Kubernetes.
4. **DaemonSets:** These enable Kubernetes users to deploy an instance of a pod to every node in the cluster.
5. **Labels:** All Kubernetes resources can be associated with labels to help identify and distinguish resources serving different purposes (e.g., pods hosting different applications).
6. **Volumes:** These are abstractions that allow the storage used by a container to be managed independently of the container. Volumes are made accessible by mounting them in a container.

### 6.1. Fundamentals

The current implementation opts for a point-to-point communication model (cf. control flow communication medium). It allows for more straightforward and explicit communication between two parties, making it more suitable for the deliberate routing decisions that consider data locality. The gRPC framework is chosen to support the communication between different components. The containers used to perform the steps, inspired from the microservice-oriented architecture, are long-lived, and each can process multiple units of data. This reduces the overhead in performance incurred by creating and deleting containers constantly throughout the execution of a workflow. A clear distinction between different aspects of the communication between components is made:

1. The components communicating with each other adhere to a particular contract or interface.
2. The actual execution is handled by the logic implementing the contract/interface.
3. The communicating parties have to employ compatible serialisation/deserialisation protocols to exchange messages.
4. The transport aspect encompasses both fundamental mediums (memory, disk, network) and abstractions on top of those (such as the HTTP protocol).

By leveraging the long-lived nature of the containers, the proposed implementation also attempts to reuse established connections to remote machines (connection pooling). The TCP protocol requires three network round-trips to perform the three-way handshake. If the protocol on top of TCP uses SSL to secure the connections (e.g., HTTPS), more network round-trips are required to negotiate and establish a secure connection. If the connection pooling is not enabled, the time to establish connections increases with the number of connections. For example, if many small data units flow through the system, the time for establishing connections can represent a significant percentage of the total execution time of the workflow. With geographically distributed resources, the performance cost of network round-trips becomes more significant with the increase in the physical distance between hosts, thus making connection pooling even more critical.

### 6.2. Orchestrator

**Figure 4:**
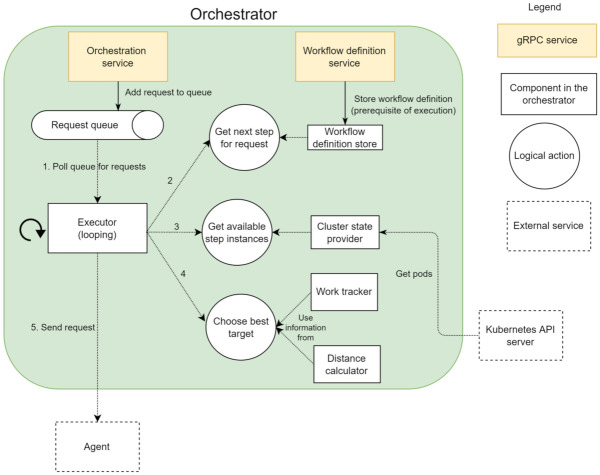
An overview of the orchestrator component.

A single instance of the orchestrator (Figure 4) is deployed, and it is for all the components in the cluster through a Kubernetes service. The single-replica strategy is needed because the central orchestrator is a stateful service that keeps the state in memory. Setting up a multi-replica orchestrator is beneficial for performance, scalability, and resilience. However, it also requires external solutions to store, share, and synchronise the state among the replicas. A workflow consists of a sequence of processing steps. Each step specifies a name that uniquely identifies the processing logic of the step. The orchestrator uses the names of steps to find pods hosting the specified processing logic through Kubernetes labels. In addition to the name, data source and data sink identifiers are also part of step specification. The data source and sink identify the data storage solutions where the input and output data should be retrieved and stored, respectively. A single workflow can utilise multiple storage solutions, such as cloud, edge, and shared storage (cf. data communication medium).

Registering and retrieving workflows is done through a service, **workflow definition service**, using **workflow definition store** and exposed by the central orchestrator. The orchestrator exposes another service, **orchestration service**, responsible for orchestrating the execution of the workflow registered through the workflow definition service. The execution of the workflow is driven by the availability of data in the system, as opposed to a task-driven execution approach [46]. The orchestrator is responsible for notifying the pods hosting the logic necessary to process the new unit of data according to the workflow definition. The communication between the orchestrator and pods hosting steps is asynchronous with respect to the actual execution of the step. The step pod does not wait for the step execution to finish before returning to the orchestrator. The **work tracker** component stores a mapping between a request and the step executing the request. The orchestrator reads this mapping to determine what is the next step to invoke. In parallel, the work tracker also keeps a counter of active computation requests for each pod. When a request arrives, it is put in a queue. This allows a response to be sent back to the caller without waiting for the finishing of the processing of the data, which can potentially be a long-running operation. The **executor** component retrieves requests from the queue and starts a background thread to process the request, allowing it to process multiple requests in parallel quickly. The **cluster state provider** component, using Kubernetes API Server, provides a list of all the pods in the cluster that can perform the desired processing logic. Finally, the agent extends the central orchestrator and handles the orchestration at a local and individual step level. It handles the communication with other components on behalf of the compute step.

### 6.3. Request Routing

Request routing discussed earlier is a multi-objective problem. For the proposed implementation, request routing uses the distance and current load as inputs. Data localisation captures information about the host (either storing the data or running a pod) and the zone. The zone is a general term meant to capture groups of hosts near one another. When routing a request through the orchestrator, a unit of data with a specified data localisation must be paired with a pod that can process it, with a potentially different data localisation.

One input to the routing decision algorithm is the distance between the localisation of the data to be processed and the localisation of each pod. A large number indicates the data transfer to the pod is likely to take longer. The distance between the same hosts is considered to be zero. For all other cases, the orchestrator constructs a N×N matrix hosting the distances between each of the *N* zones (assuming there are *N* zones in total in the system). We presume that the distance between hosts within the same zone is higher than zero but lower than the distance between two different zones. The orchestrator uses the **distance calculator** to calculate the distance (see Figure 4) and the work tracker component to get the number of concurrent requests on each pod.

**Figure 5:**
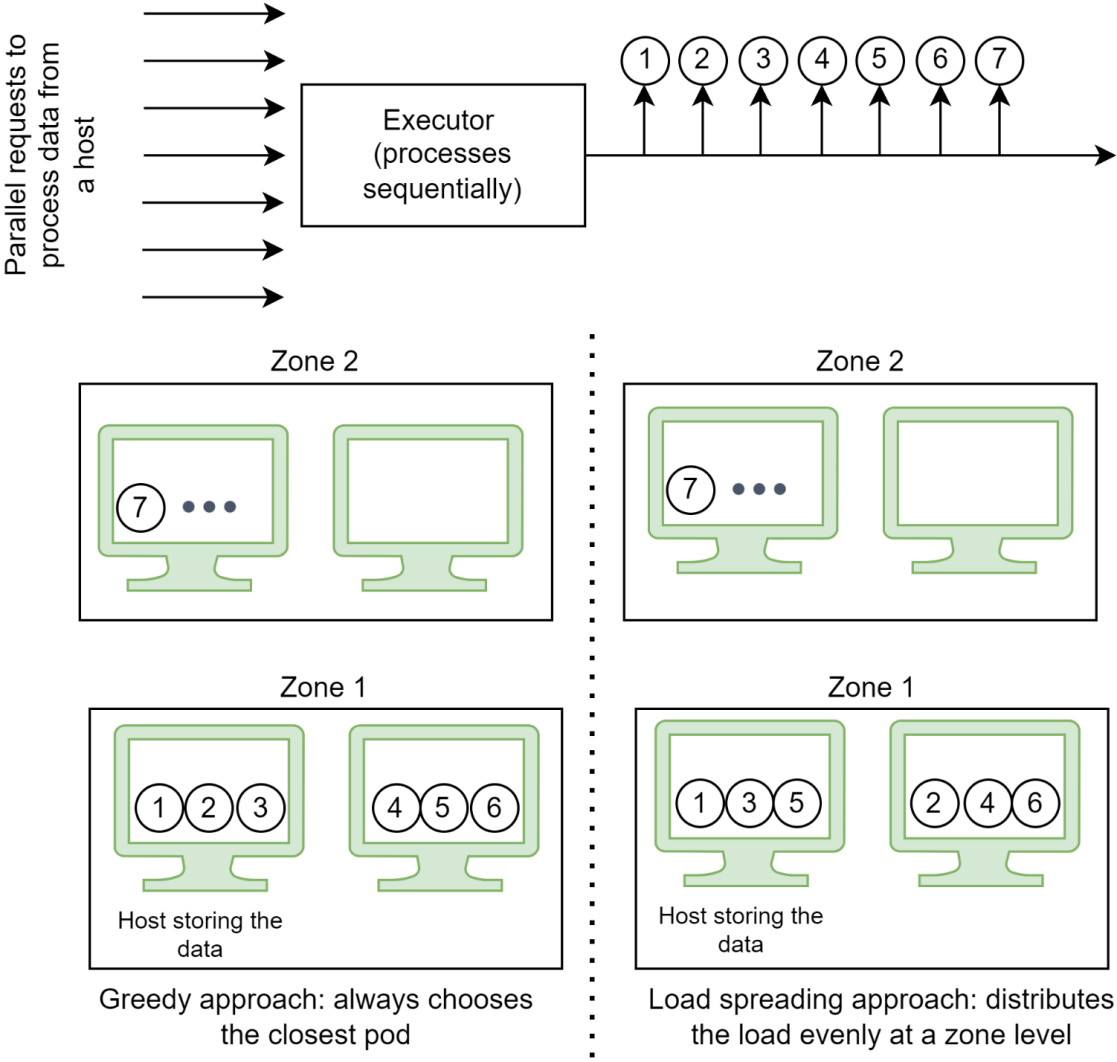
Routing priority for the greedy and load spreading algorithms.

We propose two flavors of the selection algorithm. The first one is the greedy approach: (i) the pods with several active connections higher than a configurable number (three by default) are eliminated, to avoid overloading a particular pod due to the uneven load balancing introduced by the data locality preferences, (ii) from the remaining pods, the one with the shortest calculated distance is selected (greedy selection), and (iii) if no pod remains, the request is added back into the queue to be processed at a later time. The second approach is slightly modified and focuses more on spreading the load among the available resources. Instead of always choosing the closest pod, this variant spreads the load evenly at a zone level and falls back to a different zone when the load on the pods passes a configurable threshold (three by default). The zone with available resources closest to where data are stored is chosen. Given seven requests to route, the order in which each strategy leverages the available resources is presented in Figure 5. Both algorithms exhibit the valuable characteristic of leveraging the full set of resources available in the continuum in a prioritised order to reduce the cost and latency introduced by data transfer while at the same time avoiding overloading a particular set of pods.

### 6.4. Framework Agent

Since the framework agent is logically coupled with a compute step, the two are always deployed together by leveraging the sidecar deployment pattern [47]. The agent and the compute step are separated in different containers, and they can communicate efficiently via local network calls within the host. With pods being the atomic unit Kubernetes can manage, this deployment model guarantees that each pod hosting a compute step container also contains an agent container.

The agents implement a service to expose a communication endpoint for the orchestrator. The request from the orchestrator contains the metadata needed by the data source to find the data the step should process, a unique identifier for the request, and the identifier of the data adapter to be used as a data sink. Upon receiving the request, the agent will perform the following operations:

1. Forward the metadata to the data adapter specified in the request from the orchestrator.
2. Once the data adapter ensures that the data are available at a path the compute step container can access, the compute step is invoked with the path to the file containing the data it needs to process.
3. For each output emitted by the compute step, the framework agent will instruct the sink data adapter to register the output.
4. Once the sink data adapter returns the metadata necessary to identify the newly added data, it is sent, together with the initial request, back to the orchestrator to notify that new data are available.

The agent keeps track of the number of concurrent requests being executed, allowing only a configurable number of requests to be executed in parallel.

### 6.5. Data Adapter

In the current implementation, data adapters only work with files (i.e., data retrieved from the storage solution is stored in a file, and only uploading data from a file to the storage solution is supported). Data adapters are deployed using the DaemonSet concept in Kubernetes. One pod for each type of storage adapter is deployed to every node of the Kubernetes cluster. The DaemonSet choice assumes the number of (possibly different) types of storage adapters is limited, and, thus, the overhead of running one pod of each type is negligible. Different storage solutions can be integrated, and the associated adapters can be added to extend the solution’s capabilities.

The distributed storage system leverages the local storage of every node in the cluster to store the data processed by the workflow. Besides reading and writing data, the interface also captures the localisation information about the data as part of the communication. Hard linking is used to leverage further the advantage provided by data locality. Hard linking is a faster operation than copying, and thus it further reduces the time spent on moving data between directories. The volumes are based on directories in the underlying node file system. Even though different directories are mounted as different volumes in pods, the resolution of hard links is delegated to the node file system. This allows the hard links to cross volumes mounted in different pods. By contrast, symbolic links operate by attempting to resolve a particular path, so they cannot cross different volumes unless all pods mount the same volume under the same path. A **data master** was added as a standalone component to separate data handling from the orchestration components. The data master is responsible for keeping track of the location of files stored in the proposed implementation for the distributed storage. The data master component could potentially be extended to offer more functionalities (e.g., data lineage, sharing the same data across multiple workflows, and others). The data master runs in a single pod in the cluster, made available through a service. The state of the data master is stored in memory.

The proposed solution for the distributed storage system only offers basic functionalities and lacks most of the features other alternative solutions provide (for example, data replication and redundancy, security, and resilience to failures). However, in some cases, it is possible that results from the processing steps only need to serve as input for the next step and do not have strict requirements for how the data should be stored. For such cases, the presented solution can be used as the data transfer medium, thus benefiting from an efficient means to fully leverage the data locality functionality. Within a single workflow, it is possible to use multiple storage solutions.

### 6.6. Example Flow

**Figure 6:**
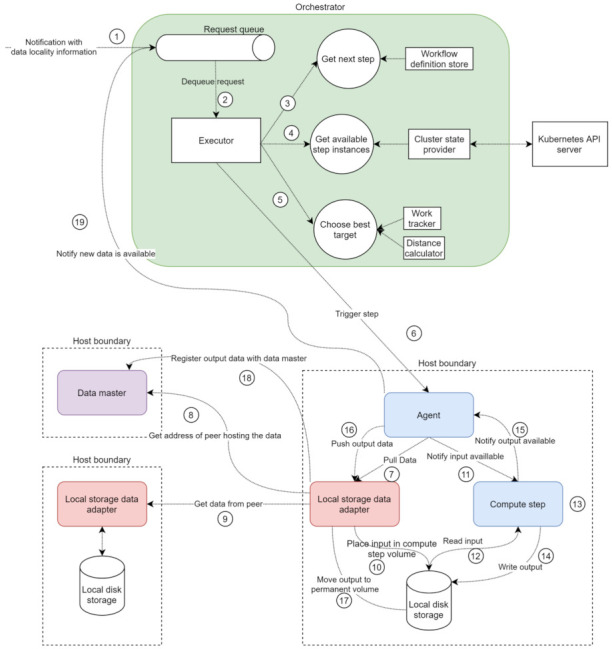
An example request flow in detail.

An example request flow is depicted in Figure 6. When a request is placed in the queue, the orchestrator is notified about available new data (1). The executor then retrieves the request (2) and starts a background thread to process the request. The first step in the processing is to determine which step should be invoked for the current notification (3), based on the request and the workflow definition. Once the step is determined, the cluster state provider provides all the available pods to perform the required step (4). Based on the current load and distance to the data to be processed, one of the available options is chosen (5), and the request is sent to the appropriate agent (6).

Once the agent receives the notification, it sends a request to the correct data adapter, based on the received data (7). If the file is not available locally, the local storage adapter will send a request to the data master (8) to determine where the file that needs to be processed is stored and will then send a request to download the data from the respective peer (9). After (8–9), or if the file is available locally, the local storage data adapter places the data in the volume the compute step can access (10) and responds to request (7). The agent then notifies the compute step that data are available (11), and the compute step reads the data (12), performs the processing logic (13), writes the output to the corresponding volume (14), and responds to the agent with the path to the output data (15). The agent sends a request to the local storage adapter to register the newly available data (16). The local storage adapter moves the new data into the permanent storage folder (17) and notifies the data master about the new data (18). The agent notifies the orchestrator that new data are available (19), and the process is repeated for all units of data flowing through the system.

## 7. Evaluation

Through a series of experiments, we compare: (i) our approach with Argo Workflows in order to analyse the run-time performances, and (ii) different configurations for the proposed solution with one another, for analysing individual aspects in isolation. The test environment is set up on the Microsoft Azure cloud using only Infrastructure-as-a-Service offerings (virtual machines and networking capabilities). The test environment is Standard D2s v3 (two vCPUs, 8GB memory) virtual machines, provisioned in three different Azure regions (EastUS, WestEurope, and NorthEurope) to mimic the geographical distribution of resources in a real cloud and edge topology. One virtual machine serves as the Kubernetes master node (and did not run additional pods). In addition to the master node, two virtual machines are configured as Kubernetes worker nodes in each region. The lightweight K3s (https://k3s.io, accessed on 8 November 2021) distribution of Kubernetes is manually installed on each virtual machine. All machines are part of the same cluster.

We use an example workflow to evaluate the architecture and implementation of the systems, which contains four sequential steps, each accepting a file as input, randomly shuffling the bytes, and writing the shuffled result as output. The motivation for choosing an artificial workflow is the ability to capture and describe the behaviour of the solution in terms of universally applicable measures (e.g., bytes for data size). In contrast, a workflow processing particular data types (such as images) captures the specific behaviour better. However, the conclusions are harder to generalise because the data type in specific cases is more restrictive in terms of size and data characteristics. For the first three steps, every machine in the cluster runs one instance of each step type. The final step is run only on machines in the EastUS region, simulating a step that can only run on cloud instances in a real scenario. Both the central orchestrator and the data master components are deployed on machines in the WestEurope region. Two additional supporting components are used when running the experiments. The load generator is responsible for injecting data into the cluster and notifying the orchestrator when data are available. The load generator creates files containing random bytes and triggers workflows for these files. The size and number of files to be injected into the cluster are configurable. The load generator also supports injecting data into two regions in parallel. The telemetry reader is responsible for gathering data on the execution of the workflow (e.g., time spent in different components, the quantity of data transferred between regions, and load spreading). The data are retrieved using the Jaeger API and is exported to CSV files, which are later analysed through Python/Jupyter notebooks.

The choice of the testing environment is motivated by the main focus of the paper that is the impact of data locality on a geographically distributed computing setting. The proposed cloud setup is capable of creating a geographically distributed system by provisioning virtual machines in different regions and integrating them into a single system. There are key differences from using a real IoT/Edge/Cloud setup, such as hardware heterogeneity (different CPU capabilities and even architectures, I/O throughput, network characteristics). However, to better isolate the benefit of data locality from other factors, the cloud setup is sufficient, as it reduces the entropy introduced by a real world setup. Changing the experimental setup either at hardware level or any upper level may result in different results, to be demonstrated and discussed further in the following subsections; however, we consider that the conditions simulated in the test setup are sufficiently generic to be applicable to a wide-range of real world scenarios. We address the primary parameters (data size and contention rates) that could influence the benefits introduced by the data locality and leave out the parameters that could play a secondary role.

### 7.1. Comparison with Argo Workflows

The example workflow presented earlier is run with four different file sizes/counts, both on Argo Workflows and the proposed solution, for comparing run-time performance. We implement the workflow using the Argo workflow definition language. The data communication medium between the steps is cloud storage (Azure BlobStorage), with one instance provisioned per region. The steps can read input data from any region but write output data to the region they are running in (e.g., a step in WestEurope can retrieve data from NorthEurope, but it always writes the output to WestEurope). Argo orchestration components (e.g., Argo Server) are deployed to worker nodes in the WestEurope region, similar to the proposed implementation.

Files of different sizes are uploaded manually to the cloud storage in WestEurope and NorthEurope region, and workflows are triggered from a client running outside the cluster, with these files as inputs. In terms of data locality, we evaluate two different approaches. First, no data locality is captured in the workflow definition, allowing the steps to be assigned to nodes anywhere in the cluster. Second, using the node selectors to limit where step pods are instantiated, the processing is kept within the same region (e.g., files originating from WestEurope were to be processed in WestEurope as much as possible). By default, the orchestration component of Argo reacts to changes in step states (e.g., a step has finished) once every 10 s. This default value is unsuitable for workflows processing small amounts of data quickly, and for these experiments, we change it to one second, which is the minimum recommended value. The exact configuration used to deploy Argo Workflows for the experiment is available in the GitHub repository of the solution.

**Figure 7:**
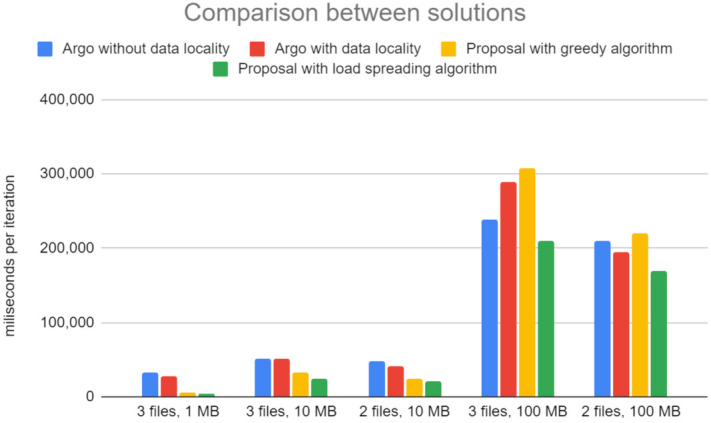
Performance comparison between the proposed solution and Argo Workflows.

Figure 7 presents the average running time of a workflow, given different data sizes and numbers of files processed in parallel. The X-axis denotes the number of files used in each of the two edge regions, WestEurope and NorthEurope, along with their size. For example, “3 files, 1 MB” indicates that three files of 1 MB each are passed through the workflow from both WestEurope and NorthEurope in parallel (six files in total). The Y-axis is the time spent on the execution of the workflow, measured in milliseconds. The numbers on the Y-axis are averaged from several iterations. The results show a low variance between different iterations. The chart compares the numbers from four runs of the same workflow: (i) in Argo, both with region-level data locality and without data locality, and (ii) in our solution, with the two flavors of the routing algorithm, greedy and load spreading.

From experiments, we can observe the following:

1. In the case of processing small data chunks that take little time to process, the proposed solution outperformed Argo workflows by a factor of five.
2. As the data size grows, the time spent on executing the logic of the step increases, and the benefit of data locality reduces.
3. Data from the second case (three files, 10 MB) show that using data locality does not affect the case of Argo workflows. Furthermore, in the fourth case (three files, 100 MB), using data locality in the workflow has a detrimental effect on performance.
4. The solutions that leverage data locality attempt to perform the work close to the data, while the other solutions spread the load evenly across the available machines. The gathered telemetry indicates that the steps of the example workflow are significantly slower when multiple instances are scheduled on the same host, as they are competing for resources.
5. Considering that there are two worker nodes available in each region, processing three files in parallel results in at least two of the files to be processed on the same node when data locality is enabled. This load distribution significantly offsets the reduction in data transfer time.
6. When processing only two files of data sizes of 10 MB and 100 MB, the load can be better spread on the testbed topology (two files can be processed in parallel on the two host machines available in each region), and the benefit of data locality can be observed.
7. On all cases, however, our proposed solution with the load spreading algorithm proves faster to execute the example workflow. However, the benefits vary, depending on the data size, from 500% (for the three files, 1 MB) to roughly 20% (for the two files, 100 MB case).

### 7.2. Evaluation under Different Configurations

A series of experiments are run with different configurations of the proposed solution to better understand the effect and behaviour of particular aspects in isolation. The time spent during the execution of the workflows is split into three categories:

(i) **Data** is referring to the time spent handling the necessary data movement (downloading the input data for a step, uploading the outputs, and moving data between directories on the node file system).
(ii) **Compute** is referring to the time spent executing the logic of the processing step.
(iii) **Control** is referring to the time spent in the orchestration component and calculated as a difference between the total execution time of a step measured by the orchestrator and the time spent on the other two categories.

**Figure 8:**
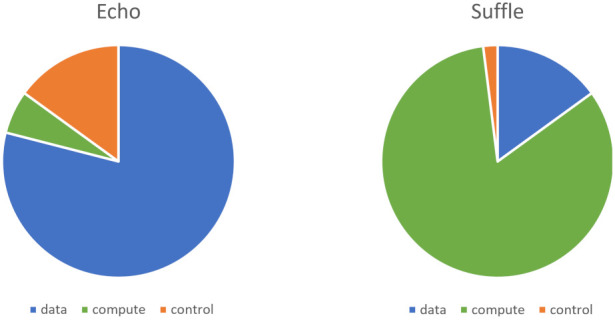
Time distribution for same workflow, but different steps: echo (**left**) and byte shuffle (**right**).

Optimising individual areas does not always translate into significant improvements in end-to-end performance. In some cases, optimisations in one area may degrade performance for the other areas. In the example workflow, most of the time is spent on the compute category, especially on larger data sizes. A different step implementation, which only reads the input and writes it to the output directly (echo functionality), is used for the following experiments. Under the same conditions, the two implementations cause a significant difference in the distribution of time. Figure 8 presents the distribution of where time is spent on average for a single-step processing 10 MB of data and showcases the significant difference between the two-step implementations.

**Figure 9:**
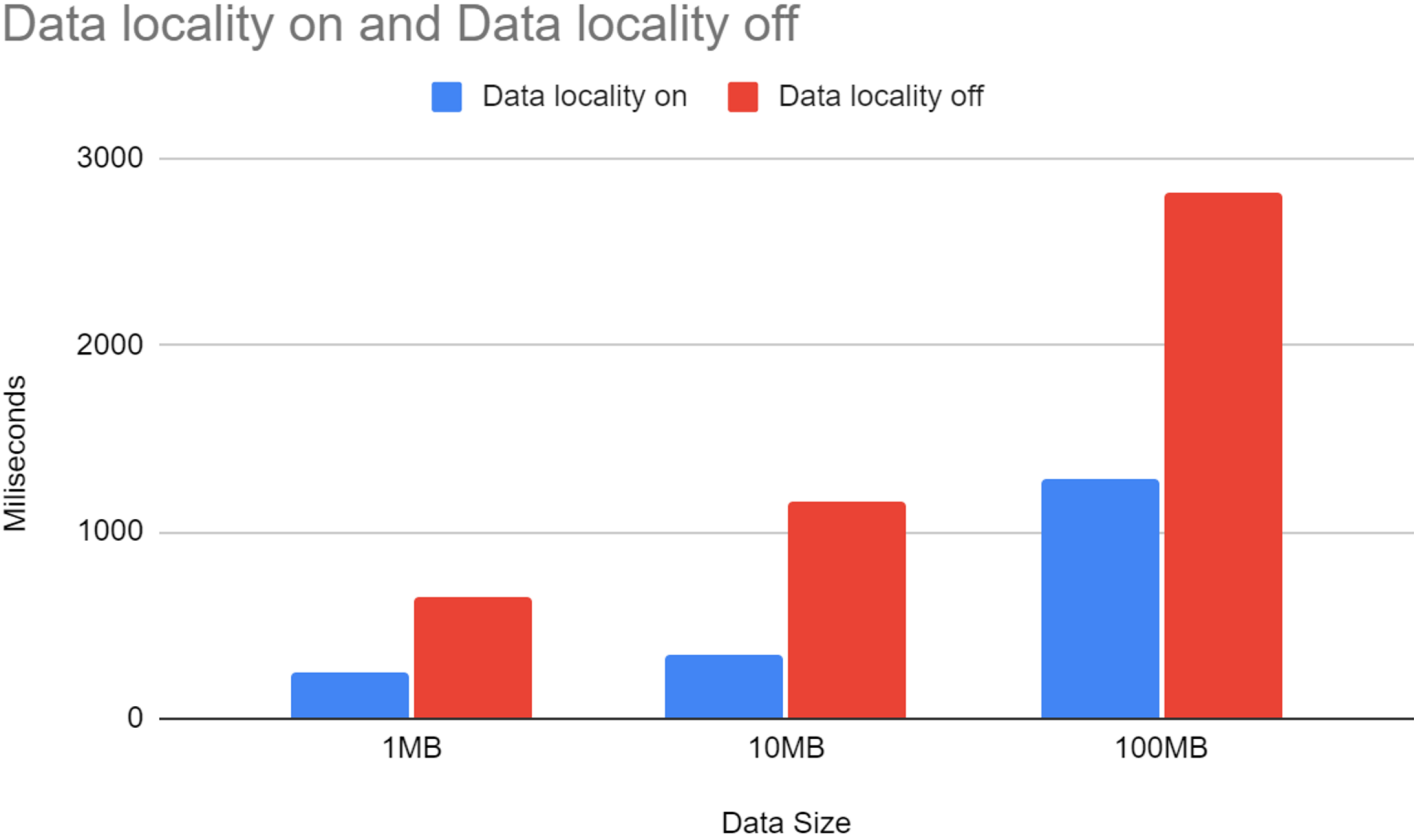
Average time spent on transferring data with and without data locality.

Regarding data handling, we analyse locality-aware routing, hard linking, and load spreading across the Computing Continuum. To study the effect of the locality-aware scheduling in isolation, a configuration allowing the orchestrator to skip the locality-aware routing and rely instead on reaching individual step instances through a Kubernetes service is used. By default, Kubernetes services use a round-robin routing algorithm, thus spreading the requests between all available instances, regardless of their physical localisation. Figure 9 shows a significant difference in average time spent for transferring data between machines, with data locality significantly reducing the time spent. The X-axis represents three different experiments. We pass files of 1 MB, 10 MB, 100 MB through the workflow, respectively. These sizes are reasonable when taking into account the scale of IoT/Edge setups. For example, given 1000 devices generating 100 MB per minute each, it suddenly becomes 100 Gb per minute in the entire system. The Y-axis represents the average time spent on moving data (in milliseconds). We observe that the more routing decisions in data are transferred over more considerable distances (e.g., from WestEurope to EastUS), the higher the step’s overall execution time. This implies that data locality is more beneficial for systems spread over more expansive areas.

An experiment comparing the performance of hard linking against the copying data indicated that hard linking executes in constant time. In contrast, time spent copying data increases with the size of the data. However, the performance benefit is negligible at the end, especially under the assumption that the size of the data directly influences the execution time of the processing step. For load spreading across a Computing Continuum, tuning the example workflow with configurations where data is produced in a single zone at increasing rates showed that the proposed solution spreads the load across the available resources. Prioritising step instances, in the order of host storing the data, host in the same zone as the host storing the data, host in the next closest zone, and host in the EastUS zone, results in bandwidth utilisation savings, especially for cases when the data locality information captures the exact host storing the data. The greedy solution is more likely to perform better when it comes to bandwidth savings, as it weighs data locality higher than the load spreading. However, it may perform poorly in terms of execution time for resource-intensive cases, as presented in experiments with Argo workflows.

Regarding the control time, we focus on long-lived containers and connection re-use. The significant difference between Argo and the proposed solution in the cases where small and frequent data units need to be processed is due to the overhead introduced by Argo using ephemeral containers. Apart from the cost of instantiating the container, observations made during the experimentation indicate that a significant portion of the time is spent starting up the application after the container is created. This observation is made because the first run of a workflow after deploying the Kubernetes cluster was significantly slower than any subsequent run. For the example workflow, with three files of 1 MB each as input, the first run took roughly 18 s while the subsequent runs took around 5 s.

We compare two configurations of the agent to measure the effect of connection re-use in the agent component: (i) in the first configuration, the connection re-use is not enabled; thus, a new connection for each message to be transmitted should be created, and (ii) in the second configuration, a single connection is re-used for all the messages. The observations indicate that while there is a performance gain in isolation (on average, 30 milliseconds per message for the first case and 10 milliseconds per message on the second case), the impact is not noticeable on the end to end latencies for the considered data sizes. However, more frequent events and lower data sizes could potentially make connection pooling a relevant optimisation.

The observations made throughout the experiments indicate that there are several variables on which the benefit of the proposed approach depends:

1. The nature of the processing steps: The resource contention caused by multiple steps running on the same machine can influence the routing algorithm’s optimal configuration controlling the balance between load spreading and data locality.
2. Distance and connection speed between the resources: A topology spreading over a wider area benefits more for the data locality-aware routing.
3. Frequency and processing duration of events: The biggest improvement is obtained by leveraging long-lived containers for frequent and small events.

## 8. Conclusions

This article proposed a novel architecture and a proof-of-concept implementation for container-centric big data workflow orchestration systems. Our proposed solution enables the orchestration components to consider data locality, quantified using a flexible model that accounts for the physical distance between hosts spread across the Computing Continuum. Our solution is better suited for processing small and frequent data units by leveraging long-lived containers re-used to process multiple units. Furthermore, it extends the ideas behind isolating processing steps in separate containers to address the data management aspect of big data workflows. As such, the logic needed to interact with data management systems is encapsulated in containers, providing the same benefits as for processing logic (technology agnostic solution, isolation, and lightweight). The communication between components is enabled by an efficient and contract-based remote procedure call framework. Finally, a set of experiments are executed to compare the proposed solution against Argo workflows and to study individual aspects in isolation.

Overall, the proposed solution improved the performance and reduced bandwidth usage for container-centric big data workflows while maintaining a good separation of concerns and reducing complexity for the framework’s users. The optimisations proved the solution better suited for environments where cloud and edge resources are leveraged together. Separating the interaction with data in a dedicated component, thus advancing toward a better separation of concerns, also facilitated the implementation of data locality as a built-in feature of the framework. This further proves the usefulness of creating small and isolated components in a distributed system. The proposed solution is far from feature-parity with the other existing solutions. However, it highlights potential areas of improvement that do not deviate significantly from the fundamentals of the existing architectures, making it possible to integrate the presented ideas and findings into them. While limited in scope, the conducted experiments are representative of the challenges the solution attempts to address. The experiments also highlighted that the benefit provided by the solution could vary significantly based on different factors. The instrumentation provided with the software solution can facilitate the same kind of performance analysis under the conditions relevant for each use case.

Future work includes extending the solution in multiple directions: (i) Simple workflow definitions were supported in the current approach to investigate potential performance improvements, but most real use cases require complex constructs. In this context, a few examples include supporting direct acyclic graph-structured workflows, processing steps to receive input from more than one data source, and supporting aggregation over multiple data units. (ii) Both data and processing logic are isolated in different components, and the workflow definition language uses these components as building blocks. A potential direction is creating a marketplace-like ecosystem where data and processing logic are exchanged between parties in the form of such components. (iii) A central piece of the proposed solution is the routing algorithm that considers load and data locality. Further improving the heuristic and adding more dimensions (such as matching node capabilities) can lead to better results. (iv) The solution could be extended to support stream processing; in addition, avoiding using a disk to transfer the data to be processed is also desirable in such cases. (v) Finally, there are two possible scaling bottlenecks in the proposal. First, the platform could benefit from creating and deleting processing steps dynamically to meet the current demands. Second, the orchestrator and the data master components are single instance applications, becoming a bottleneck under a high load and needing horizontal scaling.

## Abbreviations

The following abbreviations are used in this manuscript:

DAG	Directed acyclic graph
HDFS	Hadoop file system
IoT	Internet of things
RDMA	Remote direct memory access
